# Engineering of *Saccharomyces cerevisiae* for the accumulation of high amounts of triacylglycerol

**DOI:** 10.1186/s12934-021-01640-0

**Published:** 2021-07-27

**Authors:** Simon Arhar, Gabriela Gogg-Fassolter, Mojca Ogrizović, Klavdija Pačnik, Katharina Schwaiger, Mia Žganjar, Uroš Petrovič, Klaus Natter

**Affiliations:** 1 Institute of Molecular Biosciences, NAWI Graz, University of Graz, Humboldtstrasse 50/II, 8010 Graz, Austria; 2 Department of Molecular and Biomedical Sciences, Jožef Stefan Institute, Ljubljana, Slovenia; 3 Department of Biology, Biotechnical Faculty, University of Ljubljana, Ljubljana, Slovenia

**Keywords:** Oleaginous yeast, Neutral lipid, Lipid droplet, Lipid storage, Metabolic engineering

## Abstract

**Background:**

Fatty acid-based substances play an important role in many products, from food supplements to pharmaceutical products and biofuels. The production of fatty acids, mainly in their esterified form as triacylglycerol (TAG), has been intensively studied in oleaginous yeasts, whereas much less effort has been invested into non-oleaginous species. In the present work, we engineered the model yeast *Saccharomyces cerevisiae*, which is commonly regarded as non-oleaginous, for the storage of high amounts of TAG, comparable to the contents achieved in oleaginous yeasts.

**Results:**

We investigated the effects of several mutations with regard to increased TAG accumulation and identified six of them as important for this phenotype: a point mutation in the acetyl-CoA carboxylase Acc1p, overexpression of the diacylglycerol acyltransferase Dga1p, deletions of genes coding for enzymes involved in the competing pathways glycogen and steryl ester synthesis and TAG hydrolysis, and a deletion of *CKB1*, the gene coding for one of the regulatory subunits of casein kinase 2. With the combination of these mutations in a *S. cerevisiae* strain with a relatively high neutral lipid level already in the non-engineered state, we achieved a TAG content of 65% in the dry biomass. High TAG levels were not only obtained under conditions that favor lipid accumulation, but also in defined standard carbon-limited media.

**Conclusions:**

Baker's yeast, which is usually regarded as inefficient in the storage of TAG, can be converted into a highly oleaginous strain that could be useful in processes aiming at the synthesis of fatty acid-based products. This work emphasizes the importance of strain selection in combination with metabolic engineering to obtain high product levels.

**Supplementary Information:**

The online version contains supplementary material available at 10.1186/s12934-021-01640-0.

## Background

Most of the global fatty acid (FA) demand is currently covered by extraction from plant species that store high amounts of FA in the form of triacylglycerol (TAG) in their seeds or in the pulp of their fruits. The advantages of low costs and relatively easy scalability of these agricultural sources are compromised by factors such as the dependence of quantities and qualities of harvests on weather conditions, slow growth, seasonal peaks at harvest times instead of continuous supply, and controversial points of view on the genetic engineering of plants, mainly due to concerns regarding the release of genetically modified organisms in many countries.

These disadvantages have led to efforts to develop biotechnological processes for the production of FA. In many of these efforts algae and yeasts were used, resulting in a large number of strains with high yields and titers for TAG or FA. In yeasts, most of the work was focused on the engineering of the so-called oleaginous yeasts, a group of species with no close evolutionary relationship, but defined by their ability to store more than 20% TAG in their biomass [1]. The best-characterized of these yeasts is *Yarrowia lipolytica* and many metabolic engineering strategies to further increase the TAG content of this species have been tested, resulting in TAG contents of up to 80%, but mostly between 60 and 70% of the cell dry weight. All these engineered strains bear one or several mutations in the neutral lipid (NL) synthesis pathway. In addition, the effects of enhancing the supply of the main substrate and cofactor, acetyl-CoA and NADPH, and of reducing the flux into competing pathways were investigated (see [2] for a comprehensive recent review). Progress has also been made in the engineering of other non-conventional yeasts, although the lack of fast and efficient methods for the genetic engineering of these yeasts hampers more rapid advancement.

*S. cerevisiae* is regarded as a non-oleaginous yeast but recent work of He et al., who characterized a wild-type strain of *S. cerevisiae* with more than 20% TAG in its biomass [3], suggests that the differentiation into oleaginous and non-oleaginous yeast species, which is based on a phenotypic state that is determined by complex genetic architecture, is inadequate. In addition, its amenability to genetic modifications, its non-pathogenicity and the vast knowledge about its physiology, biochemistry and cell biology make baker's yeast a preferred microorganism for biotechnological applications. Although lipid metabolism and NL storage of *S. cerevisiae* have been characterized in detail over the recent decades [4–7], only few studies have addressed its potential as a platform for lipid production. For example, Kamisaka et al. investigated the effect of overexpression of the gene encoding diacylglycerol acyltransferase (Dgat), *DGA1*, and of an allele resulting in an N-terminally truncated variant of Dga1p, in a strain background that had a deletion of *SNF2*, the gene coding for a subunit of the SWI/SNF chromatin remodeling complex. With this combination, they obtained a lipid content of ca. 45% [8]. Because ATP citrate lyase (Acl) was considered to be one of the determinants of an oleaginous phenotype [9], Tang et al. constructed a strain that heterologously expressed murine Acl in an *idh1∆ idh2∆* deletion background. Whereas no changes were observed during growth, the strain accumulated ca. 20% more FA than the wild-type in the stationary phase [10]. In another study, the endogenous pathway of FA synthesis was targeted directly, by overexpression of *ACC1*, coding for acetyl-CoA carboxylase, and *FAS1* and *FAS2*, which encode the two subunits of the type I FA synthase (FAS) complex in yeast. This strategy resulted in ca. 17% TAG in the biomass, a four-fold increase in comparison to the wild-type [11]. An engineering approach that also targeted *ACC1*, in combination with the overexpression of *PAH1*, coding for the phosphatidic acid (PA) hydrolase, and of *DGA1* resulted in a strain that accumulated ca. 13% TAG in its biomass. With the additional deletions of TAG lipases, a steryl ester (SE) synthase and a peroxisomal long-chain FA importer, a TAG content of ca. 25% was achieved [12]. The most heavily engineered strain was constructed in an attempt to combine Acl expression with respiratory metabolism, thereby mimicking the physiology and the lipid metabolism of an oleaginous yeast. To this aim, Acl was expressed in a strain that had a deletion of *IDH1* and of the three genes coding for pyruvate decarboxylase, *PDC1*, *PDC5* and *PDC6*, which required evolutionary adaptation to restore growth in glucose media. In addition, the pentose phosphate pathway was upregulated to increase the supply of NADPH and the cell was engineered for excretion of FA instead of their storage as TAG. With this strain, a titer of ca. 33 g/L excreted FA and a yield of 0.1 g/g glucose, corresponding to ca. 30% of the theoretical yield, was obtained [13, 14].

These studies suggest that the TAG content of *S. cerevisiae* can be increased dramatically by genetic engineering. On the other hand, some of these engineered strains accumulated less lipids than the wild-type strain D5A, which was shown to be an oleaginous strain of baker's yeast [3]. In the current study, we investigated the effects of a combination of a starting strain with already high TAG content with genetic engineering strategies, with the aim to maximize TAG production and storage in *S. cerevisiae*. This work resulted in a strain with a TAG content that is comparable with engineered oleaginous yeasts.

## Results

### The TAG content in *S. cerevisiae* strains covers a broad range

**Fig. 1:**
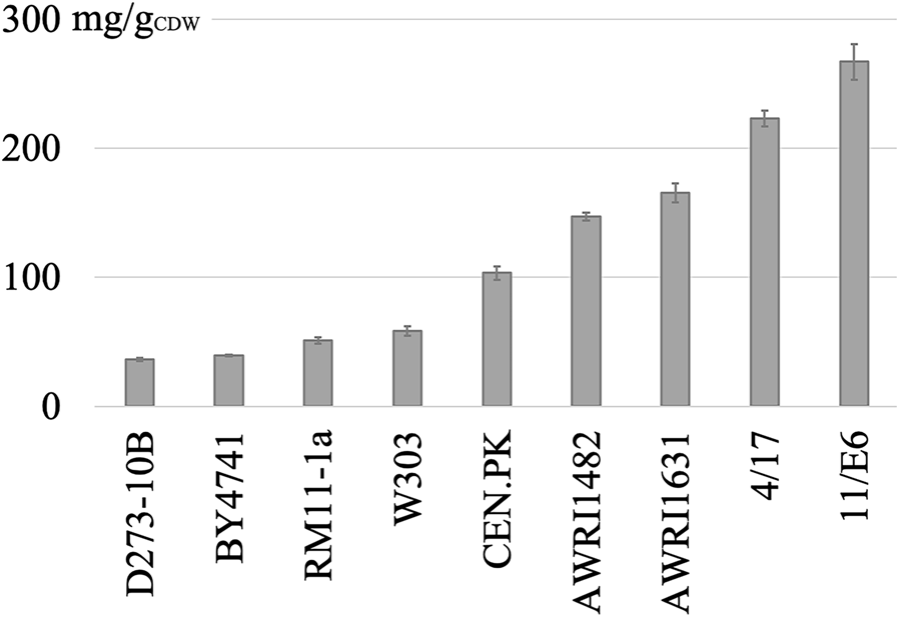
TAG content in yeast wild-type strains after cultivation for 96 h in lipid accumulation-inducing medium. The first five strains are commonly used laboratory strains; AWRI1482 and AWRI1631 are natural strains, the latter being a stable haploid; 4/17 and 11/E6 are segregants derived from sporulations of AWRI1631xCEN.PK and AWRI1631xBY4741 diploids, respectively. TAG was extracted and quantified after cultivation for 96 h under nitrogen-limited conditions (MM/N-lim1)

In this study, we aimed at the engineering of a *S. cerevisiae* strain with high TAG content. To determine an appropriate wild-type background for the accumulation of high TAG levels, we first analyzed seven strain backgrounds after growth in a medium that stimulates lipid accumulation (MM/N-lim1). As shown in Fig. 1, the TAG content of yeast strains covers a relatively broad range, with only 37 mg TAG per g cell dry weight (CDW) in the strain with the lowest content, D273-10B, and with 165 mg/g_CDW_ in AWRI1631, the strain with the highest lipid content. To test the possibility whether even higher TAG contents might be obtained through crossing, AWRI1631 was mated with the second-best haploid strain with regard to TAG accumulation, CEN.PK. The segregant with the highest lipid content that was obtained from this procedure (see Methods), 4/17, accumulated 223 mg TAG per g_CDW_ (Fig. 1). In addition, we tested several segregants that were obtained from a cross between AWRI1631 and BY4741 [15]. One of these segregants, 11/E6, accumulated 267 mg TAG per g_CDW_, corresponding to a more than seven-fold higher TAG content than in D273-10B, the strain with the lowest content.

Further crossings of the segregant 4/17 with W303 and of 11/E6 with CEN.PK, however, resulted in progeny with lower TAG content (data not shown). In addition, the segregant 11/E6 had lower biomass yields than most other strains shown in Fig. 1, resulting in a lower lipid titer than the one obtained with 4/17 (Additional file 1: Figure S1). Therefore, 4/17 was used for the subsequent genetic manipulations to further increase the TAG content.

### Engineering of the TAG pathway

**Fig. 2:**
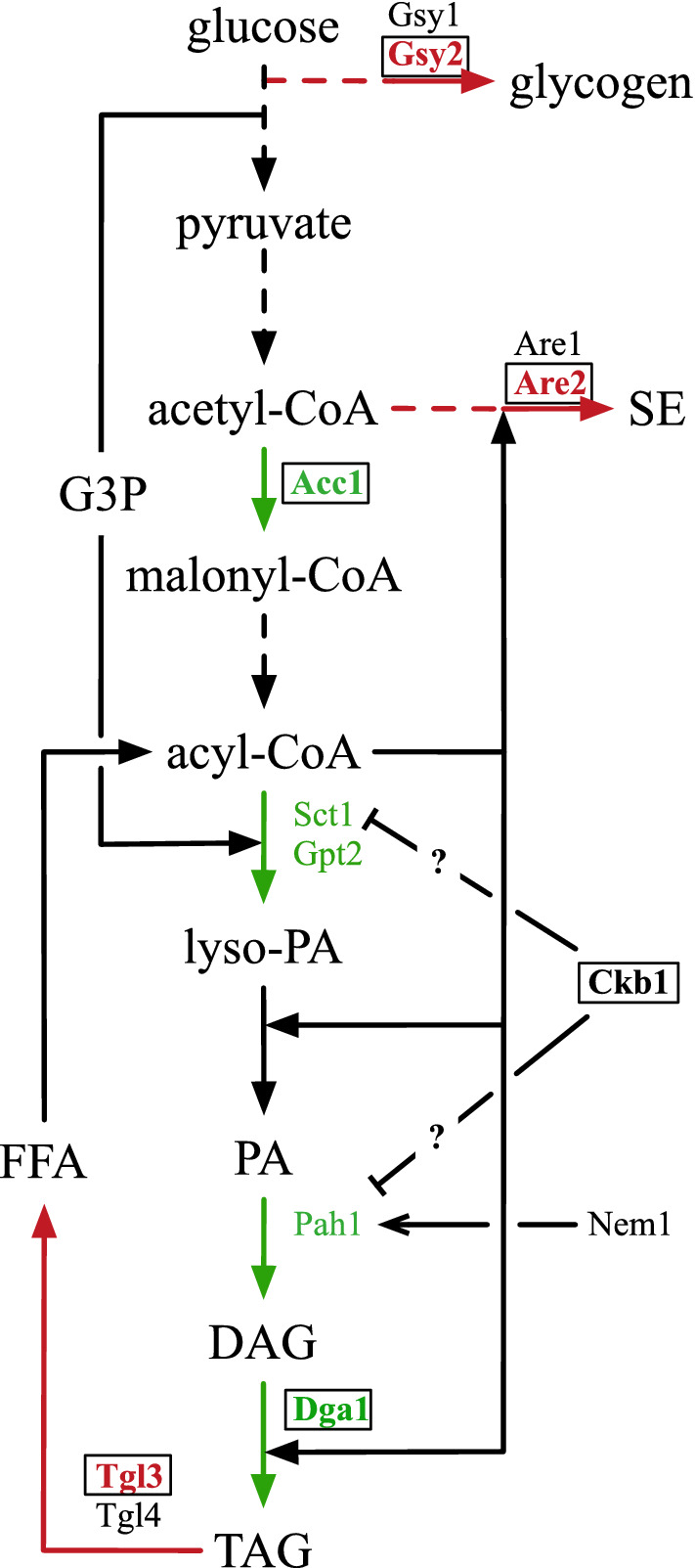
Metabolic pathway of TAG synthesis from glucose. Reactions that were investigated in this study and had a positive effect on TAG accumulation are colored. Pathway reactions that were upregulated are shown in green. For Ckb1p, which is not encoding a metabolic enzyme, we assume a repressing effect on the activities of Sct1p and possibly Pah1p through phosphorylation, in the case of Pah1p antagonistic to the Nem1p/Spo7p phosphatase complex (see main text). Competing pathways, which were partially blocked, are depicted in red. The mutated variant of Acc1p, together with an overexpression of *DGA1* and the deletion of *CKB1, GSY2, ARE2* and *TGL3* (framed reactions) resulted in a strain with 65% TAG content. *Dashed lines* several reactions, *straight lines* single reactions, *G3P* glucose-3-phosphate, *FFA* free fatty acids, *DAG* diacylglycerol

Because several enzymes of the TAG synthesis pathway have been shown to be subject to extensive regulation, the flux through this pathway is probably not controlled by a single rate-determining step. To assess the quantitative contributions of some of the reactions leading to TAG, we introduced single mutations in the segregant 4/17, followed by lipid analysis. Successful modifications are summarized in Fig. 2.

#### Mutations in Acc1

**Fig. 3:**
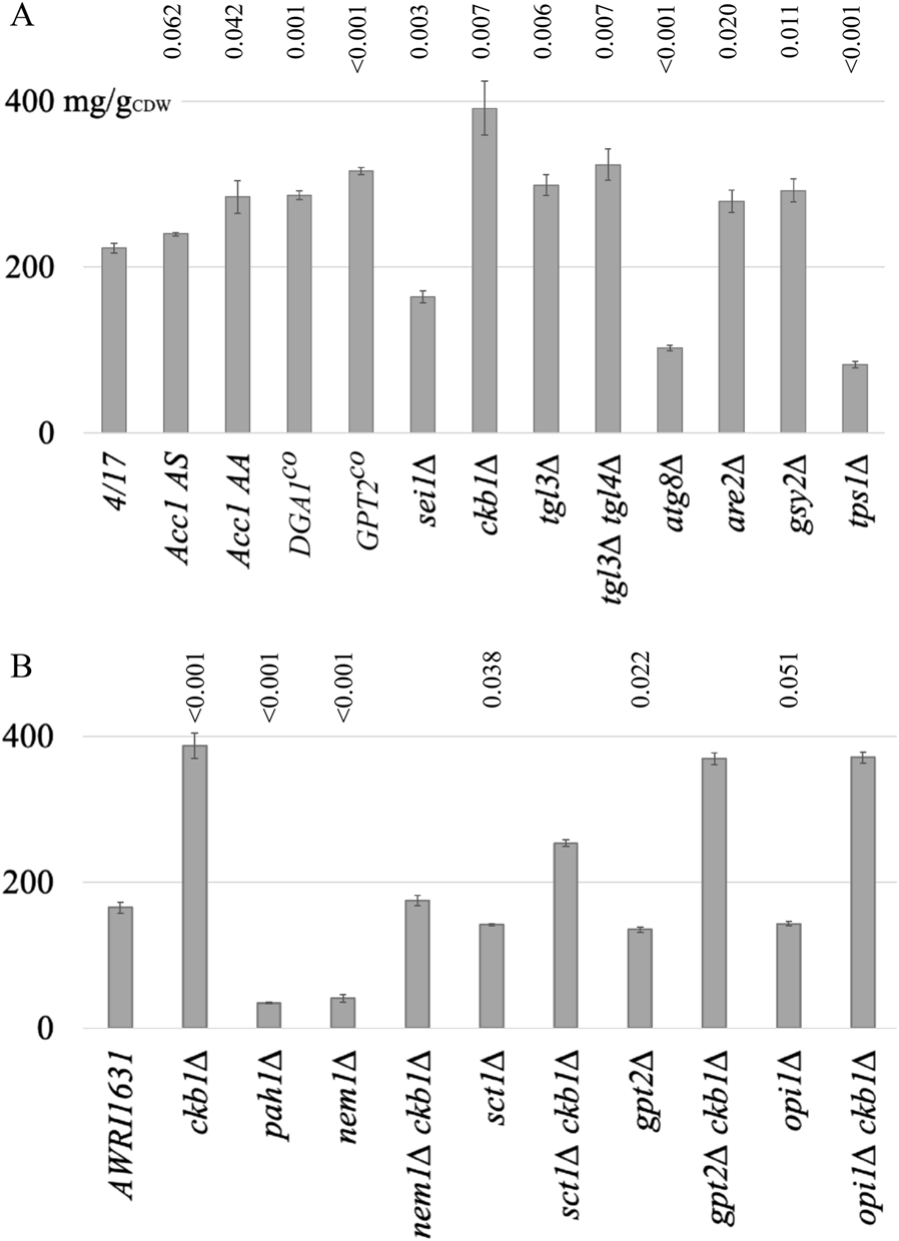
Effects of single mutations on TAG content. **A** TAG content in the segregant 4/17 bearing mutations in the product pathway or in a competing pathway. Acc1 AS indicates an alanine at position 1157 and a serine at position 1257 of Acc1p. The mutation of position 1257 to alanine in this strain results in Acc1 AA. *DGA1*^*co*^ and *GPT2*^*co*^ indicate the overexpression of codon-optimized variants of these two genes. **B** TAG content in strains with deletions of genes that encode possible substrates of CK2 and the respective double mutants in the *ckb1*∆ background. The p-values above the columns are derived from a comparison of the single mutants with the wild-type parental strain. In **B** All p-values for the comparison of the double mutant strains with their respective single deletion parental strain are < 0.001. All strains were cultivated for 96 h under nitrogen-limited conditions (MM/N-lim1)

An example of a strong regulation on the post-translational level is Acc1p. The activity of Acc1p is inhibited by phosphorylation of a serine at position 1157 through the AMP-activated kinase, Snf1p. A mutation of this serine residue to alanine results in higher enzymatic activity, resulting in higher TAG levels [16]. This serine at position 1157 is also present in the AWRI1631 strain background. In addition, the ORF of *ACC1* in AWRI1631 bears a single nucleotide variation at position 3769, which results in an alanine at position 1257 of the protein, instead of a serine, which is present in most other *S. cerevisiae* strains. Although no phosphorylation of S1257 has been reported, we constructed mutants of the segregant 4/17 with an alanine at position 1157 and either alanine or serine at position 1257. The mutant with alanine residues at both positions accumulated the highest amount of TAG (Fig. 3A), indicating that the residue at position 1257 contributes to the regulation of the activity of Acc1p.

#### Overexpression of acyltransferases

Overexpression of Dgat, the final enzymatic activity in the TAG pathway that transfers an acyl chain from acyl-CoA to diacylglycerol, is included in most strategies to engineer microorganisms for high levels of TAG. Increased TAG content through higher Dgat activity requires a feedback mechanism that results in a stimulation of de novo FA synthesis. One possible explanation is the negative feedback loop that inhibits Acc1p and/or the FAS complex through binding of the end product of FA synthesis, palmitoyl-CoA [17–20] because the high Dgat activity would deplete the cellular palmitoyl-CoA pool.

Because no regulatory mechanisms are known that control the activity of Dga1p, the Dgat in *S. cerevisiae*, we designed a codon-optimized variant of the *DGA1* gene under the control of a strong constitutive promoter, to achieve high protein levels. As expected, this strategy resulted in an increase in TAG accumulation, from 223 to 287 mg/g_CDW_ (Fig. 3A).

Several acyltransferases contribute to the synthesis of TAG. If the abovementioned reduction of the palmitoyl-CoA pool causes the increased lipid synthesis in the *DGA1* overexpressing strain, other acyltransferases should have a similar effect. The first step in glycerolipid synthesis is catalyzed by glycerol-3-phosphate acyltransferase (Gpat) and two genes in *S. cerevisiae*, *GPT2* and *SCT1*, encode this activity. Sct1p was shown to utilize palmitoyl-CoA efficiently. However, its overexpression has been reported to cause a reduced growth rate and a lower biomass yield [21]. Therefore, we expressed a codon-optimized variant of *GPT2* under the control of a strong constitutive promoter. This modification caused an increase of the TAG content from 223 to 316 mg/g_CDW_ (Fig. 3A).

#### Deletion of a regulatory subunit of casein kinase 2

In a screening of the yeast deletion collection for strains with a phenotype called 'supersized lipid droplets', Fei et al. have identified several mutants with this unusual lipid droplet (LD) morphology [22]. In the culture of such a mutant, many cells have been observed to bear only one large LD instead of many small ones as is the case in wild-type cells. The authors have found that this phenotype correlates with high levels of PA and speculated that PA might stimulate LD fusion. In fact, the LD morphology in these mutants appears very similar to oleaginous yeasts during cultivation under NL-inducing conditions, when TAG is stored in only one or two large LDs [23]. Therefore, we wanted to investigate, whether the supersizing of LDs in *S. cerevisiae* results in a higher NL content. We selected two genes, *CKB1*, encoding a regulatory subunit of casein kinase 2 (CK2), and *SEI1*, the seipin homologue in yeast. As shown in Fig. 3A, these mutations had opposite effects on the storage of TAG. Whereas the deletion of *SEI1* caused a strong decrease, the *ckb1∆* mutant had the highest TAG content of a single mutant that we obtained in this study. This result suggested that it is not the supersizing phenotype per se that is important for high levels of TAG but another role of Ckb1p.

The strong impact of the deletion of *CKB1* on TAG accumulation was not specific for the strain used in this study, but was also observed for other strain backgrounds (Additional file 1: Figure S2). To investigate this effect in more detail, we attempted to construct several double-knockout strains to identify putative interaction partners. CK2 has been reported to phosphorylate the PA hydrolase Pah1p [24], which is known to have a strong influence on the synthesis of TAG. Furthermore, the two proteins with Gpat activity, Gpt2p and Sct1p, are phosphorylated, making them possible substrates for CK2. Although the transcriptional repressor Opi1p is not known to play a role in lipid storage, it was included in this experiment because it has been shown to be a substrate for CK2 [25]. Whereas we successfully constructed the single deletion mutants for *PAH1, GPT2*, *SCT1* and *OPI1* and the double-knockout strains of the latter three in combination with *ckb1*∆, we failed to obtain a *ckb1∆ pah1∆* double mutant in several experiments. The dissection of 50 tetrads of a heterozygous *CKB1/ckb1∆ PAH1/pah1∆* diploid strain revealed a pattern of viable segregants that is typical for a synthetic lethal phenotype of the double mutation. Therefore, we chose an alternative approach and constructed the double mutant *nem1∆ ckb1∆*. Nem1p is a putative catalytic subunit of the Nem1/Spo7 phosphatase complex that acts on several phosphorylation sites of Pah1p, including residues phosphorylated by CK2 [24]. The deletions of *PAH1* and of *NEM1* resulted in similarly strong effects on TAG accumulation, with a reduction of 79 and 75%, respectively (Fig. 3B). The additional deletion of *CKB1* in the *nem1*∆ background restored TAG storage to a similar level as in the wild-type parent. Due to the complex phosphorylation pattern of Pah1p it is not possible to conclude to which extent the effect of the *ckb1*∆ mutation in this background is caused by an activation of Pah1p. On the other hand, the result clearly indicated that Pah1p is not the only factor responsible for the strong phenotype of a *ckb1*∆ strain because the deletion of *CKB1* in the *nem1*∆ background resulted in a several-fold increase of the TAG content. For the other three genes, we found moderately reduced TAG levels in the single deletion strains. In the *opi1∆ ckb1∆* and *gpt2∆ ckb1∆* double mutant strains, the TAG content was restored to the level of the *ckb1*∆ single mutant, suggesting that there is no interaction between Ckb1p and Opi1p or Gpt2p that affects TAG synthesis. The deletion of *CKB1* in the *sct1∆* background, however, resulted only in a partial restoration of the *ckb1*∆ phenotype (Fig. 3B). This result allows for the interpretation that Sct1p is negatively regulated by CK2. Such a conclusion is supported by microscopic data, showing that the strong supersizing phenotype of the *ckb1*∆ mutant was reverted to a wild-type-like LD morphology upon deletion of *SCT1*. The deletion of the *SCT1* paralog *GPT2*, on the other hand, showed no such effect. In the *nem1∆ ckb1∆* mutant, we observed a slight reduction of the supersizing phenotype, indicating that a reduced phosphorylation status of Pah1p contributes to the strong TAG accumulation phenotype of the *ckb1∆* strain (Additional file 1: Figure S3).

Finally, to investigate the possibility that Acc1p is a substrate of Ckb1p, we deleted *CKB1* in four strains that had been mutated to bear all possible combinations of serine or alanine residues at the positions 1157 and 1257 of Acc1p. The analysis of these four stains and their *CKB1* parental strains showed that the effect of the *ckb1*∆ deletion on the TAG content is independent of the *ACC1* allele, suggesting that serine residues at the positions 1157 and 1257 of Acc1p are not phosphorylated by Ckb1p (Additional file 1: Figure S4).

### Down-regulation of competing pathways

Several pathways and processes might interfere with high TAG levels. We investigated four of these competing reactions, namely SE synthesis, glycogen storage, TAG hydrolysis and LD autophagy.

#### TAG hydrolysis

TAG lipase activity would not only reduce TAG yields through the consumption of the product but also through the released FA, due to the inhibition of FA synthesis after their activation to acyl-CoA. Although TAG hydrolysis activity might be low when the cells are stimulated for its storage, the assumed role of TAG in the FA remodeling of phospholipids suggests a continuous turnover of this metabolite, with synthesis and degradation in parallel. Indeed, the deletion of the gene coding for the TAG lipase Tgl3p resulted in a clear increase in TAG content, which was 34% higher than that of the wild-type strain. The deletion of a second gene coding for a TAG lipase, *TGL4*, in the *tgl3*∆ strain background did not result in a further increase of TAG storage under nitrogen-limited conditions (Fig. 3A). Moreover, the biomass yield of the double mutant was lower than that of the *tgl3∆* single mutant (Additional file 1: Figure S1), resulting in lower lipid titers.

These two enzymes, Tgl3p and Tgl4p, are the major TAG lipases acting on cytosolic LDs. During stationary phase and under nitrogen-limiting growth conditions, however, a subset of LDs is incorporated into the vacuole and subsequently degraded by different enzymes [26]. It is not known whether a loss of LD autophagy would result in a higher TAG content under nitrogen-limited conditions. Because no protein is known that is specifically responsible for LD autophagy, but not involved in other autophagic processes, we deleted *ATG8*, resulting in a mutant in which autophagy is absent. This mutation, however, resulted in a strong decrease of the TAG content by 47% (Fig. 3A), confirming a similar effect that was reported for carbon-limited conditions [27]. Therefore, even though the degradation of TAG via lipophagy is prevented in such a strain, the absence of autophagy seems to have a negative effect on the TAG content of yeast.

#### Steryl ester synthesis

The synthesis of ergosterol and SE requires acetyl-CoA, the cofactors ATP and NADPH and, in the case of SE, acyl-CoA, making this pathway a direct competitor for TAG synthesis. Due to the essential role of ergosterol, down-regulation of the pathway is prone to lead to reduced robustness of the strain when the rate for ergosterol synthesis drops below a critical level. Because this level is not known and the exact regulation of a long pathway is difficult, we only targeted the synthesis of SE, the storage form of excess ergosterol. As shown in Additional file 1: Figure S5, the strain used for this study, segregant 4/17, accumulated the same amount of SE as the CEN.PK parental strain, but less than the AWRI1631 parental strain. We deleted the gene coding for the major sterol acyltransferase, Are2p, and analyzed the resulting strain for its storage lipid content. The TAG content of this mutant increased by 25% (Fig. 3A). The additional loss of the second sterol acyltransferase activity, Are1p, and hence the inability to store ergosterol as SE (Additional file 1: Figure S5), did not result in a further increase of the TAG content but in lower final biomass, similar to our observations for the TAG lipase double mutant strain (Additional file 1: Figure S1).

#### Glycogen synthesis

In a similar manner as TAG accumulation, glycogen synthesis and storage are induced during cultivation under nitrogen-limiting conditions [28]. We have previously shown that both *Y. lipolytica* and *S. cerevisiae* compensate for the loss of glycogen storage by accumulating more storage lipid, although this effect was weaker in baker's yeast. However, the strains used in that study had been derived from CEN.PK [29] and we assumed that the properties of the segregant 4/17 with regard to glycogen storage and the response to its loss could be different. Indeed, this strain accumulated high amounts of glycogen. We obtained a content of 183 mg/g_CDW_ after growth in nitrogen-limited medium (Additional file 1: Figure S6). Based on the assumption that a reduction of glycogen storage would result in a redirection of this carbon flux to NL synthesis, we deleted *GSY2*, coding for the main glycogen synthase activity in *S. cerevisiae*. The subsequent analysis confirmed that this mutation resulted in a clear improvement of TAG accumulation, with an increase of 35% as compared to the wild-type (Fig. 3A). Surprisingly, the glycogen content in this strain was the same as in the wild-type. Therefore, we also constructed the 4/17 *gsy1*∆ mutant. In this strain, glycogen storage was reduced to ca. 50% of the wild-type, indicating that Gsy1p is the main glycogen synthase activity under nitrogen-limited conditions (Additional file 1: Figure S6). The TAG content, on the other hand, remained unchanged (data not shown). As already observed for the other competing pathways, the complete elimination of glycogen synthesis in the *gsy1∆ gsy2∆* double mutant resulted in a decrease of the biomass yield (Additional file 1: Figure S1), but in no further improvement of the TAG content as compared to the *gsy2∆* single mutant.

In addition to glycogen, trehalose is a major carbohydrate storage form in *S. cerevisiae*. Therefore, its synthesis could also be regarded as a competing pathway for TAG synthesis. To determine the effect of the loss of trehalose synthesis on TAG accumulation, we deleted *TPS1*, the gene coding for trehalose-6-phosphate synthase. This mutation did not cause a growth defect on glucose, as it was reported for other strain backgrounds [30], but it had a strong negative effect on lipid storage, with a TAG content of only 37% as compared to the wild-type strain (Fig. 3A).

### Engineering of the acetyl-CoA and NADPH supply does not increase TAG accumulation

High rates of FA synthesis rely on the supply of high amounts of the substrate, acetyl-CoA, and the redox cofactor, NADPH. Engineering of these metabolites has been included in many engineering strategies aiming at high flux through this pathway (reviewed in [23]). To assess the effects of interventions in the supply of NADPH and acetyl-CoA, we chose several strategies. First, we overexpressed the acetaldehyde dehydrogenase-encoding gene *ALD6*. Ald6p is the native NADP^+^-dependent *S. cerevisiae* enzyme in the pyruvate decarboxylase pathway and its overexpression should result in a higher conversion rate of acetaldehyde to acetate, resulting in better availability of NADPH and in higher TAG content if this reaction contributes to the limitation of the efficiency of the TAG synthesis pathway. Second, we expressed the glyceraldehyde-3-phosphate dehydrogenase (GAPDH) from *Kluyveromyces lactis*, which has similar K_m_-values for NAD^+^ and NADP^+^, in contrast to the homologues of *S. cerevisiae*, which are strictly NAD^+^-dependent. We replaced the ORF encoding the major GAPDH of *S. cerevisiae*, *TDH3*, with a codon-optimized variant of the *K. lactis* gene *GDP1*, resulting in a partial loss of the native NAD^+^-dependent GAPDH activity and heterologous expression of *GDP1* under control of the strong *TDH3* promoter, as confirmed with a GAPDH enzyme assay (Additional file 1: Figure S7). This overexpression of *GDP1* was combined with the overexpression of a codon-optimized variant of the gene coding for NAD^+^/NADH kinase, *UTR1*, to increase the intracellular pool of NADP^+^/NADPH. From this strategy, we expected a partial switch of glycolysis from NAD^+^- to NADP^+^-dependency, resulting in better NADPH balance and in a higher rate of FA synthesis for the recycling of NADPH to NADP^+^. In addition, the reduction of NADH formation in glycolysis was expected to result in reduced excretion of ethanol because the redox cycle of glycolysis is shifted to NADP^+^/NADPH and, hence, also in higher biomass yields. Third, to improve the supply of acetyl-CoA, we constructed a vector bearing the genes coding for both the regulatory and the enzymatic subunits of ATP-citrate lyase (Acl) of *Y. lipolytica*. The heterologous expression of Acl in *S. cerevisiae* was combined with the deletion of *IDH1* to increase the intracellular level of citrate, the substrate of Acl. Finally, two expression vectors bearing one of the yeast genes encoding acetyl-CoA synthetase, *ACS1*, were constructed. Acs1p bears a putative N-terminal signal for nuclear targeting and a peroxisome targeting-like signal (-VKL) at its C-terminal end. Furthermore, a lysine that was reported to be involved in the regulation of Acs in *Salmonella enterica* through acetylation-dependent inactivation of the protein [31] is conserved in *S. cerevisiae* (K675). We tested the effects of the wild-type allele and of a sequence encoding a N- and C-terminally truncated protein with a K675R mutation (*ACS1*^*mut*^).

**Fig. 4:**
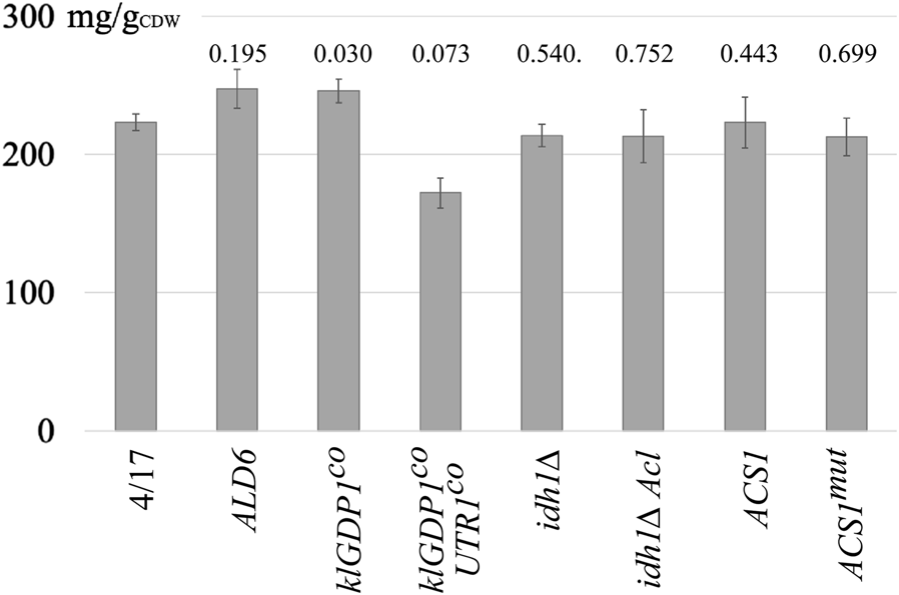
Engineering of acetyl-CoA supply and NADP^+^/NADPH balance. The figure shows the TAG content of segregant 4/17 bearing mutations with an effect on the acetyl-CoA and NADP + /NADPH balance. The numbers above the columns are p-values derived from a comparison of the mutants with the wild-type in a two-tailed t-test. The strains were cultivated for 96 h in MM/N-lim1

The results of these experiments are shown in Fig. 4. Only the expression of the NAD^+^/NADP^+^-dependent GAPDH from *K. lactis* and of the aldehyde dehydrogenase Ald6p resulted in moderate increases of the TAG content as compared to the wild-type strain. The additional overexpression of *UTR1* in combination with *klGDP1* caused not only a drop of the TAG content, but also a reduced growth rate and lower biomass yields. This was also observed for strains bearing the *idh1∆* mutation (data not shown). Neither the heterologous expression of Acl nor the overexpression of *ACS1* or its mutated variant resulted in any changes in TAG storage.

### *S. cerevisiae* mutants accumulate TAG to up to 65% of their biomass

**Fig. 5:**
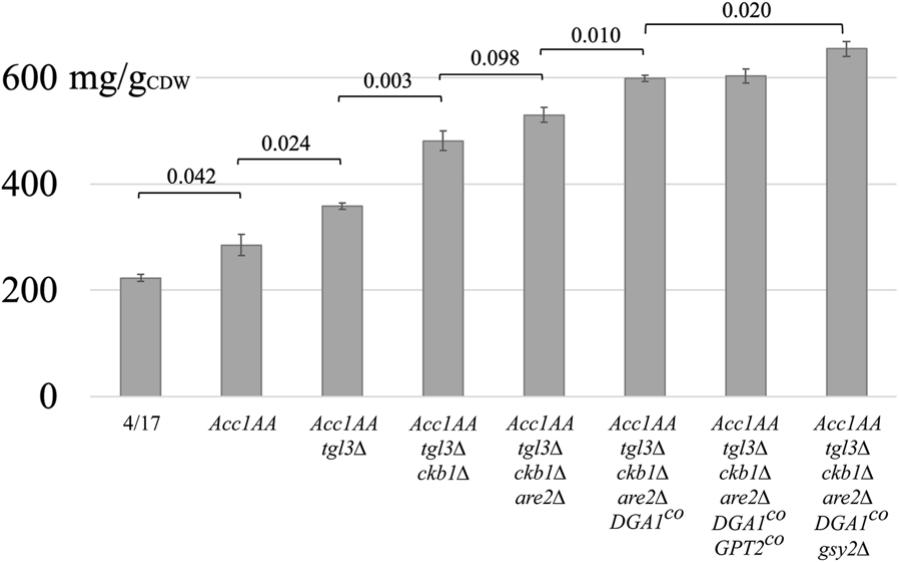
Engineering of segregant 4/17 for high TAG content. The single mutations with a positive effect were combined in one strain, resulting in a mutant that accumulated 65% TAG after the introduction of six mutations. All experiments were performed in nitrogen-limited media (MM/N-lim1). The numbers above the columns indicate the p-values calculated with an unpaired two-tailed t-test

We constructed a mutant bearing all mutations that were beneficial for TAG accumulation (Fig. 2). Starting with the wild-type background 4/17, we introduced the mutation in *ACC1* that results in the exchange of serine to alanine at position 1157. In this strain, *TGL3*, *CKB1* and *ARE2* were deleted consecutively, resulting in a strain that accumulated already more than 50% TAG in its biomass (Fig. 5), which, to our knowledge, is the highest reported TAG content for *S. cerevisiae* so far. To further increase TAG accumulation in this strain, we chromosomally integrated the codon-optimized variants of *DGA1* and *GPT2*, both under the control of a strong constitutive *TEF1* promoter. Whereas the insertion of *DGA1*^*co*^ (resulting in strain 4/17–5) induced a further increase to 60% TAG in the biomass, the additional expression of *GPT2*^*co*^ had no significant effect on TAG accumulation (Fig. 5), although this mutation had caused an increase in the wild-type background.

With regard to glycogen storage, we found that the high flux of glucose to TAG resulted in a decrease of the glycogen content from 18 to 7% (Additional file 1: Figure S6) in strain 4/17–5. To evaluate the possibility of an increase of lipid synthesis upon further reduction of glycogen storage, we constructed mutants of 4/17–5, in which either *GSY1* or *GSY2 was deleted*. The deletion of *GSY2* resulted in a reduction of the glycogen content to 22 mg/g_CDW_ (Additional file 1: Figure S6) and in a further improvement of TAG accumulation. This strain with six mutations (named 4/17–6 in the following text) contained 65% TAG in its biomass (Fig. 5). For *GSY1*, we successfully constructed the deletion mutant. However, for unknown reasons this strain had a lag-phase of several days under nitrogen-limited conditions and grew only to low cell densities. Therefore, this mutant was not further analyzed.

Importantly, the growth rate of strain 4/17–6 was only moderately lower than that of the wild-type parental strain, with a maximum specific growth rate µ = 0.34/h, as compared to µ = 0.36/h for the wild-type during growth in a defined minimal medium (MM/C-lim). In a nitrogen-limited medium that was optimized for a higher biomass yield (MM/N-lim2), both strains grew with almost the same rates as in MM/C-lim. In this medium, we obtained a CDW of 4.17 g/L for 4/17-6, as compared to 3.09 g/L for the wild-type (Additional file 1: Table S2). This value corresponds to a TAG yield of 13.6% for the mutant and to ca. 41% of the theoretical maximum yield if a complete conversion of glucose to TAG is assumed [32].

**Fig. 6:**
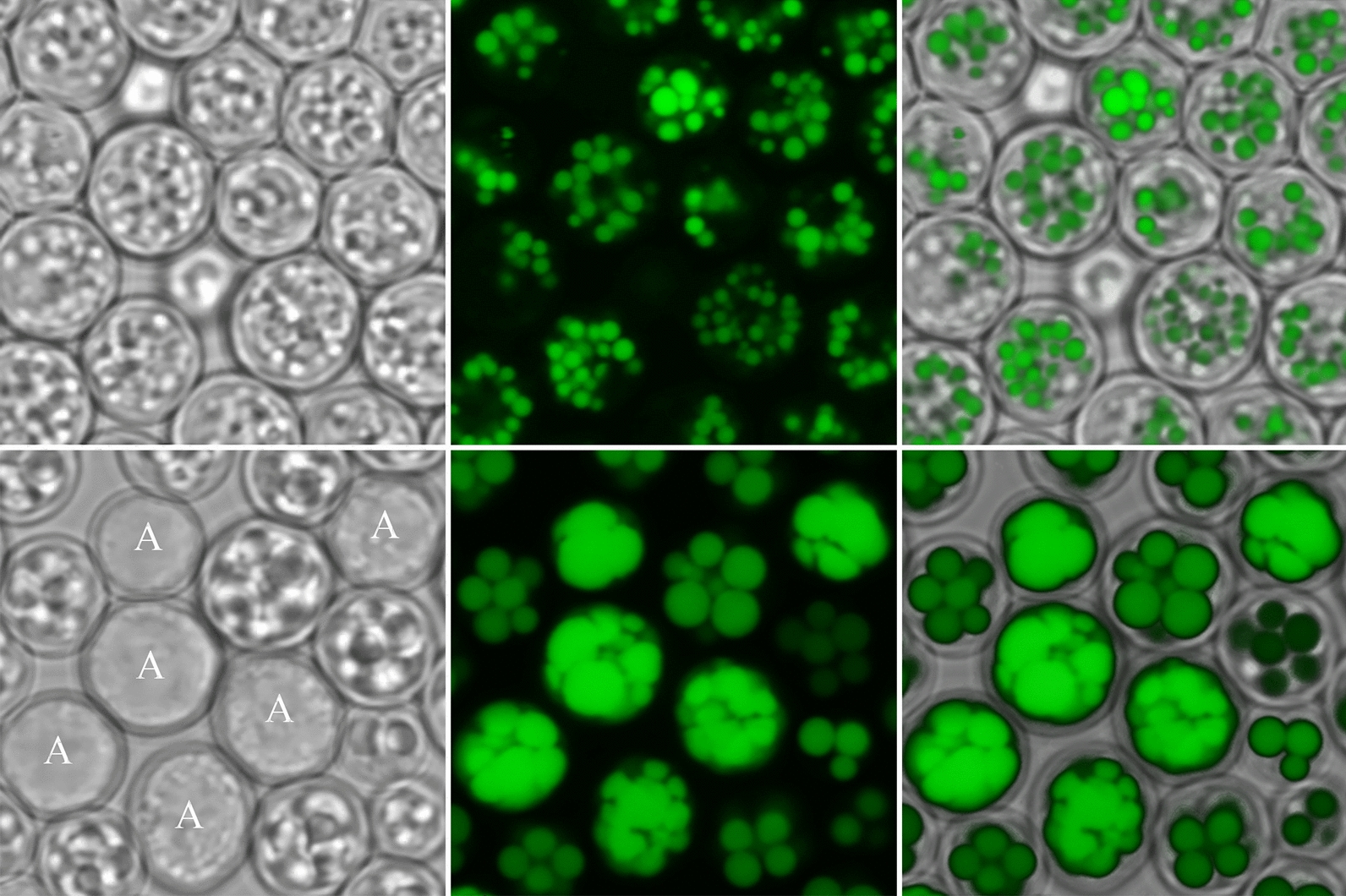
Microscopy of yeast under TAG accumulating conditions. Upper row: wild-type strain 4/17, lower row: the same strain after engineering for high TAG levels. The lipid droplets were stained with Bodipy 493/503 after growth of the strains in MM/N-lim1 for 96 h. The cells of the engineered strain in which the whole interior appears as an amorphous mass are marked with an A. The images show 20 × 20 µm sections

Microscopic inspection after cultivation under lipid accumulation inducing conditions showed that most of the mutant cells were packed with large LD. In a considerable fraction, however, the interior of the cells appeared as an amorphous mass in the transmission images, with no recognizable subcellular structures. Staining with the hydrophobic fluorophore Bodipy 493/503 showed that these cells were even more densely packed with LDs, which seemingly occupied almost the whole intracellular space (Fig. 6). Upon incubation in fresh YPD medium, these cells did not start budding within 8 h, whereas the other cells showed buds within 2–3 h, indicating that the densely packed cells were not viable any more. After plating of aliquots from nitrogen-limited cultures of the wild-type 4/17 and of the mutant 4/17–6 onto YPD plates, 98% and 75% of the cells, respectively, formed colonies.

Fatty acid methyl ester (FAME) analysis showed that the FA composition of the engineered mutant was changed. It should be noted, however, that with this method all FA, including those from phospholipids and SE, are measured. This analysis resulted in a slightly higher content of lipid in the biomass (68% FAME) than the analysis with thin layer chromatography (65% TAG), a method in which the lipid classes are separated and can be quantified individually. As expected, all FA were increased in the mutant but a clear shift to longer chain lengths, mainly from C16:1 to C18:1 was observed (Additional file 1: Figure S8). The C16/C18 ratio dropped from 1.6 to 1.1.

Although the expression of enzymes aiming at changes in the supply of acetyl-CoA and of the NADP^+^/NADPH balance did not result in higher TAG values in the wild-type (Fig. 4), we repeated these experiments in the engineered strain because the availability of acetyl-CoA and NADPH could limit a further increase of lipid synthesis when the flux is already high. In addition, we tested the effect of heterologous expression of *OIL1* from *Y. lipolytica*, which is required for the oleaginous phenotype of this yeast [33], and of the perilipin-like protein in *S. cerevisiae*, Pln1p, which was shown to stabilize LDs [34]. Although their functional role in NL storage is not fully understood, we assumed that *yl*Oil1p or Pln1p might result in a stabilization of the large LDs in the engineered strain and, therefore, in the ability of the cell to further increase the TAG content. Although we achieved a slight improvement with regard to the number of viable cells in the strains overexpressing y*lOIL1* and *PLN1*, this did not result in higher TAG contents. Similarly, the TAG content remained unchanged in the strains overexpressing one of the two *ACS1* alleles, Acl, *klGDP1* or *ALD6* (Additional file 1: Figure S9).

### Decoupling of TAG accumulation from nitrogen limitation

**Fig. 7:**
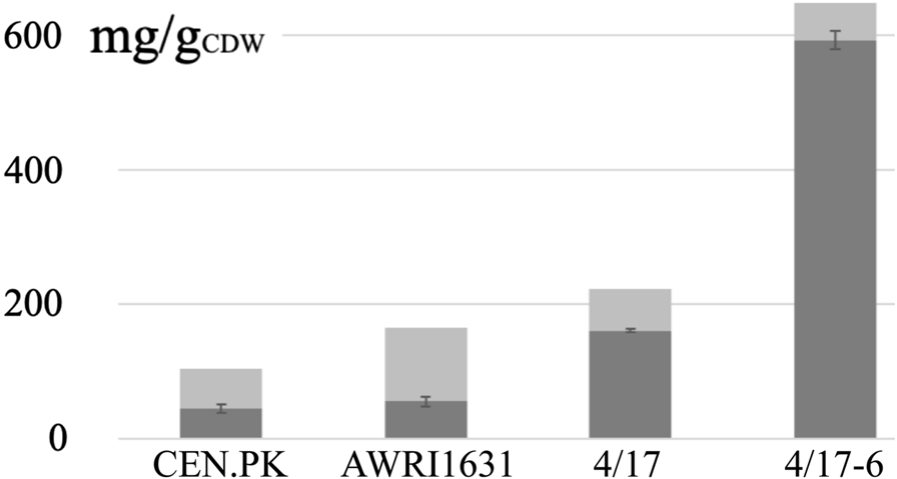
Decoupling of TAG accumulation from nitrogen limitation. The dark gray areas show TAG accumulation after growth under carbon-limited conditions (MM/C-lim). For comparison, the values for the cultivations in nitrogen-limited media (MM/N-lim1) are shown in light gray

Yeasts, like other microorganisms, accumulate TAG only under specific conditions, with a growth arrest due to nitrogen limitation being the most common such laboratory condition. In contrast, if yeasts enter stationary phase due to the depletion of the carbon source or if they grow exponentially without any nutritional limitation, only small amounts of TAG are stored. For the strain engineered for high TAG content in this study, we wanted to assess its potential with regard to the accumulation of TAG in carbon-limited media. Surprisingly, the engineered strain, when cultivated in MM/C-lim, accumulated high amounts of TAG. Our analysis showed that the mutant 4/17–6 contained 59% TAG in the stationary phase (96 h of cultivation), corresponding to 92% of the value after growth under nitrogen-limited conditions. Even the wild-type strain, 4/17 stored high amounts of TAG, ca. 72% of the value obtained after growth in MM/N-lim1 (Fig. 7). In the parental strains AWRI1631 and CEN.PK, on the other hand, the TAG contents after growth in nitrogen-rich media were only ca. 33% and 42%, respectively, of the values after growth under nitrogen limitation. Therefore, we assume that the partial decoupling of TAG accumulation from nitrogen-limitation is determined in the genomic background of 4/17 and is not a result of one or several of the mutations that were introduced. However, this behavior was only observed in defined minimal medium, whereas in the complex medium YPD the TAG contents of the wild-type and mutant dropped to 28 and 253 mg/g_CDW_, respectively (data not shown).

## Discussion

In this work we aimed at the engineering of a *S. cerevisiae* strain with high TAG content. Our initial analysis of wild-type strains demonstrated that this phenotype is highly variable in this species. One reason for the rather low levels in some commonly used laboratory strains may be that these strains have been selected for phenotypes that are not directly related to lipid accumulation. These selection procedures may have resulted in a loss of high TAG levels or even in a counter-selection against this trait. On the other hand, it remains unclear if wild *S. cerevisiae* strains in general accumulate high amounts of storage lipids or if this is an exceptional trait of the two tested strains, AWRI1631 and AWRI1482. We showed by crossing wild-type strains that the TAG content of *S. cerevisiae* can easily exceed the arbitrary benchmark of 20%. Therefore, the current classification of yeasts into oleaginous and non-oleaginous species should be scrutinized. This is supported by findings that not all wild-type strains of the so-called oleaginous yeasts, such as *Y. lipolytica*, contain more than 20% TAG in their biomass [33]. Furthermore, attempts to identify differences between oleaginous and non-oleaginous yeasts that determine this phenotype on the molecular, physiological or biochemical level have failed so far [35].

Many of the known regulatory mechanisms in the pathway leading to TAG are based on protein phosphorylation, as in the case of the acetyl-CoA carboxylase Acc1p [16, 36], the Gpat enzymes Gpt2p and Sct1p [21, 37], and the phosphatidic acid phosphatase Pah1p [38]. Our strategy included two interventions that resulted in deregulation of enzymes due to reduced phosphorylation, the mutated variant of Acc1p and the deletion of *CKB1*, coding for a subunit of CK2. CK2 has many substrates and is involved in the regulation of a large number of diverse processes. Therefore, the strong increase of the TAG content after the deletion of *CKB1* could be the cumulative effect of reduced phosphorylation of several of these substrates. With regard to the TAG pathway, our results indicate an interaction between *CKB1* and *SCT1* and that the high TAG level in the *ckb1*∆ mutant is in part caused by derepression of Sct1p. It can be speculated that this stimulation at the first step of glycerolipid synthesis, together with the almost complete loss of the ability to store excess lipids as TAG in the *pah1*∆ mutant, causes the observed synthetic lethality of the *ckb1∆ pah1∆* double mutant strain. These mutations together probably result in the strong accumulation of lyso-PA and PA, but also of other phospholipids and, hence, in the proliferation and expansion of membranes. This hypothesis is in agreement with the observation that both the overexpression of *SCT1* and the deletion of *PAH1* result in growth defects [21, 39], which might become lethal in combination.

Although the deletion of *CKB1* resulted in the highest TAG content of all single mutations, it was clearly not sufficient to fully activate the TAG synthesis pathway. Our results suggest that its full activation also requires the stimulation of FA synthesis, in our case through the mutation of Acc1p, and the overexpression of the final step, encoded by *DGA1*. Such an effect of the last step of a pathway is unexpected because it requires the activation of upstream reactions that result in an increased supply of the substrates of Dga1p, diacylglycerol and acyl-CoA. It remains unclear, whether this activation is caused by the depletion of the palmitoyl-CoA pool, which inhibits FA synthesis [17–20], or by another mechanism. Nevertheless, our results with the overexpression of the Gpat encoding gene *GPT2* support the former explanation because its overexpression alone resulted in an increase of the TAG content, whereas it had no effect in a background that was already overexpressing *DGA1* (Figs. 3A, 5).

Our experiments aiming at the down-regulation of competing pathways showed that the complete elimination of these processes is detrimental for the cells, at least under nitrogen-limited conditions. Although none of the storage metabolites—glycogen, TAG and SE—are required for viability and even the complete loss of all three pathways is not lethal [29], both double mutants, *are1∆ are2∆* and *gsy1∆ gsy2∆,* achieved lower biomass yields than the corresponding single mutants. The same was observed for the down-regulation of TAG lipolysis (Additional file 1: Figure S1). Therefore, a low flux through these pathways seems to be the better strategy than setting them to zero because unknown direct or indirect effects of the storage metabolites, their precursors or the products of their degradation might be important for cellular fitness. An indication for such unknown effects were our experiments regarding the elimination of glycogen synthesis. Based on the hypothesis that the deletion of this pathway will result in a redirection of the carbon flux to TAG synthesis, we deleted *GSY2*, which is reported to encode the major glycogen synthase activity [40]. Intriguingly, this mutation caused the expected increase of the TAG content under nitrogen-limited conditions without a simultaneous decrease in glycogen storage, refuting the assumption of a redirection of the carbon flux through elimination of glycogen synthesis. The deletion of *GSY1*, on the other hand, resulted in a reduced glycogen content, indicating that Gsy1p is the major glycogen synthase under these conditions. The TAG content of this mutant remained unchanged. If *GSY1* was deleted in the strain engineered for TAG synthesis the cells were viable in rich medium and under carbon-limited conditions, but could no longer be cultivated in nitrogen-limited media. On the other hand, the deletion of *GSY2* in this strain resulted in the expected changes, i.e. a higher TAG and lower glycogen content. Hence, in our case the deletion of *GSY2* was the most successful strategy, but this might be specific for the strain background and the mutations that we used to achieve the high TAG content.

The engineering of the supply of acetyl-CoA, the main substrate for TAG synthesis, and of the cofactor NADPH did not result in an improvement of the TAG content in the tested strain background, neither in the wild-type nor in the engineered mutant. This was surprising because similar approaches were applied successfully in other studies [10, 14, 41–43]. One possible reason is that the supply with acetyl-CoA and NADPH in the segregant 4/17 is already better than in other strains. Even in the strain with 65% TAG content, sufficient amounts of these two metabolites could be synthesized without engineering, suggesting that this strain is able to respond flexibly to increasing demands of acetyl-CoA and NADPH. This assumption is supported by other studies that reported on strains with high contents of TAG without the manipulation of pathways leading to acetyl-CoA or NADPH [2].

We were not able to increase the TAG content of our strain beyond 65% of the biomass. Nevertheless, the results of this study do not allow for a conclusion with regard to the maximum possible value that can be obtained in *S. cerevisiae* without a strong impairment of cellular viability. It has to be assumed that, under conditions of lipid accumulation to such high values, cytosolic processes are considerably slower because of the increased size of LDs at the expense of the aqueous phase. In addition to the reduced water content, an increase of the TAG content to ca. 65% of the biomass must be accompanied by a dramatic reduction of the other major biomass components, protein, RNA, glycogen and the cell wall. Furthermore, microscopic data (Fig. 6) and growth tests indicated that the viability of cells with such a high TAG content is reduced. The existence of an upper limit to TAG accumulation is supported by work with oleaginous yeasts, in which a lipid content between 60 and 70% was achieved in many studies, whereas only few authors reported higher values of up to 80% TAG content [2].

## Conclusion

*S. cerevisiae* can be engineered for the accumulation of TAG to a level that is comparable with the values obtained with engineered oleaginous yeasts. The wealth of knowledge about *S. cerevisiae*, its non-pathogenicity and the availability of many tools for rapid and efficient genetic manipulation make it a promising host for the development of processes aiming at the production of FA-based products. Importantly, future efforts will have to address the possibility to combine TAG production with second generation processes utilizing cellulose-derived biomass as substrate and engineering strategies to reduce ethanol excretion, to improve product yields in this Crabtree-positive yeast.

## Methods

### Strains and cultivation conditions

The *S. cerevisiae* strains BY4741 [44], CEN.PK 113-7D [45], W303 [46], D273-10B (ATCC 24657), RM11-1a [47], S288c [48], AWRI1631 [49], AWRI1482 and AWRI1633 (the AWRI strains were kind gifts from the Australian Wine Research Institute) were used in this study. For strain maintenance, standard YPD media (20 g/L glucose, 20 g/L peptone, 10 g/L yeast extract; + 20 g/L agar for plates) were used. If required, 200 mg/L geneticin/G418 (Calbiochem) for selection of cells bearing the *KanMX* marker or 100 mg/L nourseothricin (Werner BioAgents, Germany) for the *NatMX* marker were added after autoclaving.

Carbon-limited minimal medium, MM/C-lim, contained 20 g/L glucose, 5 g/L (NH_4_)_2_SO_4_, 3 g/L KH_2_PO_4_, 0.5 g/L MgSO_4_ and vitamins and trace elements according to [50], without inositol (carbon to nitrogen ratio C/N = 7.6). The medium was buffered to an initial pH of 5.7 with 10 mM phthalate buffer. Standard nitrogen-limited minimal medium, MM/N-lim1, was identical to MM/C-lim, except for the ammonium sulfate concentration, which was 0.2 g/L, and only 2 mM phthalate buffer, resulting in growth arrest after depletion of the nitrogen source, when the glucose concentration was still high (C/N = 189). For the determination of yields with the engineered strain with high TAG content, MM/N-lim2 with 20 g/L glucose, 0.6 g/L ammonium sulfate, 1 g/L KH_2_PO_4_, 0.25 g/L MgSO_4_, vitamins and supplements as above, buffered with 6 mM phthalate was used (C/N = 63). This ratio of carbon to nitrogen resulted in the same TAG contents as in MM/N-lim1 and in cell densities similar to those achieved in MM/C-lim. For the cultivations of the auxotrophic strains in MM/N-lim1, the auxotrophic markers were complemented with 30 mg/L adenine, 40 mg/L uracil, 40 mg/L tryptophan, 100 mg/L leucine, 30 mg/L histidine (W303); 40 mg/L uracil, 100 mg/L leucine, 30 mg/L histidine, 40 mg/L methionine (BY4741); 40 mg/L uracil, 100 mg/L leucine (RM11-1a).

Sporulation plates contained 10 g/L potassium acetate, 1 g/L yeast extract, 0.5 g/L glucose and 20 g/L agar.

For lipid analysis, cells were inoculated from plates into preculture tubes containing the same medium as the main culture. 500 mL flasks containing 100 mL medium were inoculated with exponentially growing precultures to a cell density of 10^8^ cells/L. Both the preculture and the main culture were incubated at 30 °C and 180 rpm. Culture aliquots were harvested after 96 h for the determination of CDW and for lipid extraction.

### Strain construction

To obtain a wild-type strain with high TAG content, AWRI1631 was crossed with a *MAT*α derivative of CEN.PK 113-7D. The diploid strain was sporulated on sporulation plates for four days and complete tetrads were dissected on YPD plates.

After germination of the haploids, 136 viable segregants were transferred to MM/N-lim1 plates and cultivated for three days. The LD size was assessed under the microscope and the 20 segregants with the highest score were subjected to lipid analysis after cultivation in MM/N-lim1. The strain with the highest lipid content was crossed with W303. After sporulation, haploids were obtained by the protocol for random spore analysis [51] and growth on MM/C-lim plates without amino acids, to obtain only prototrophic segregants. 96 colonies were picked and analyzed as described for AWRI1631xCEN.PK.

### Strain engineering

For the introduction of mutations with CRISPR/Cas9, the plasmid pCR_empty [29] was digested with *Not*I. The region bearing the *CEN6/ARSH4* origin, the gene for resistance to nourseothricin and the *SNR52* promoter were amplified by PCR from pCRISPR_CAS9_gRNA.GSY1 [29] and assembled with the linearized vector by Gibson assembly [52], resulting in the plasmid pCRISPR. This plasmid bears a *Not*I restriction site between the *SNR52* promoter and the gRNA. Together with a double-stranded oligo containing the recognition sequence, the *Not*I-linearized vector can be assembled by Gibson assembly to a ready-to-use CRISPR/Cas9 vector. This vector, together with a ds-oligo for the repair of the strand break and introduction of the desired mutation, was used to transform the recipient strains with the lithium acetate method [53]. The recognition sequences and sequences of the repair oligonucleotides are listed in Additional file 1: Table S1. Mutations were confirmed by Sanger sequencing. Cells that had lost the pCRISPR plasmid after growth in YPD were selected for the next engineering step.

Segregant 4/17 bears the single nucleotide variation of the AWRI1631 parent that results in an alanine at position 1257. The S1157A mutation of Acc1p in the segregant 4/17 was obtained with CRISPR/Cas9 (Additional file 1: Table S1), resulting in an Acc1p variant with alanine residues at positions 1157 and 1257. To obtain the 1157A 1257S variant, we first introduced the 1157A mutation in CEN.PK 113-7D. A DNA fragment starting ca. 200 bp upstream of the codon for 1157A and ending ca. 200 bp downstream of the codon for 1257S was amplified from this strain. The PCR product was used to transform the segregant 4/17. Positive transformants were selected on YPD plates containing 1 µg/ml soraphen A according to [16]. With this strain, the CRISPR/Cas9 approach described above was used to obtain the mutant with serine residues at both positions. All point mutations were confirmed by Sanger sequencing.

For the overexpression of *GPT2*, a codon-optimized sequence (*GPT2*^*co*^) was designed, resulting in an improvement of the gene's codon bias from CAI = 0.11 to 0.97 [54]. After DNA synthesis (Twist Biosciences), the gene was amplified with primers bearing overhangs for Gibson assembly with the linearized vector pHEYg-1. This vector was obtained from pHEY-1 [29], by insertion of a *loxP-KanMX-loxP* cassette directly upstream of the *TEF1* promoter. After Gibson assembly with *GPT2*^*co*^, this vector, pHEYg-1/*GPT2*^*co*^, bears a cassette *KanMX-TEF1*^*P*^*-GPT2*^*co*^. This cassette was amplified with primers bearing overhangs up- and downstream of the native ORF of *GPT2*. Transformation of yeast with this PCR product and homologous recombination resulted in replacement of the native *GPT2* with the amplified cassette. Correct insertion was confirmed by PCR and the *KanMX* cassette was removed by *Cre* recombinase treatment.

The same strategy as described for *GPT2* was used for the overexpression of *DGA1*, resulting in the replacement of the native *DGA1* (CAI = 0.072) with the codon-optimized *DGA1*^*co*^ (CAI = 0.86) under control of the *TEF1* promoter. The transformation efficiency was improved with a pCRISPR plasmid according to Additional file 1: Table S1.

For the heterologous expression of *K. lactis GDP1* (CAI in *S. cerevisiae* = 0.27), a codon-optimized variant of the ORF (*klGDP1*^*co*^*, CAI* = *0.85*) was cloned into the vector pHEY-1. The cassette bearing *klGDP1*^*co*^ and the *CYC1* terminator was amplified and inserted in the START region of *TDH3* with a CRISPR/Cas9-based approach (Additional file 1: Table S1), resulting in *klGDP1*^*co*^ under the control of the *TDH3* promoter.

For the overexpression of *UTR1* (CAI = 0.074*)*, pHEYg-1 was digested with *Eco*RI and *Sal*I, resulting in linearization and excision of the *TEF1* promoter. The truncated *HXT7* promoter [55] was amplified from *S. cerevisiae* genomic DNA with primers bearing overhangs for the assembly with the linearized pHEYg-1. Gibson assembly of the two DNA fragments resulted in pHEYg-2, allowing for the expression of genes under the control of the strong truncated *HXT7* promoter. pHEYg-2 was linearized and Gibson-assembled with a codon-optimized variant of *UTR1* (*UTR1*^*co*^*, CAI* = *0.82*), resulting in a vector bearing the cassette *KanMX-HXT7*^*P*^*-UTR1*^*co*^*-CYC1*^*T*^. The cassette was amplified and inserted upstream of the promoter region of *CDC19* on chromosome I by using a CRISPR/Cas9 strategy.

In some cases, a standard protocol for gene deletions was used. In short, the *loxP-KanMX-loxP* cassette was amplified with primers containing overhangs for homologous recombination upstream of the 5'-end and downstream of the 3'-end of the target ORF, resulting in START to STOP replacements of the ORF after transformation. The correct insertion of the cassette was confirmed by PCR before the *loxP-KanMX-loxP* cassette was removed by *Cre* recombinase treatment for recycling of the marker.

For the deletion of *NEM1, SCT1, GPT2* and *OPI1* in the *ckb1*∆ background the *NatMX* cassette was amplified from plasmid p4339 [56] with primers bearing the appropriate overhangs for the replacement of the complete ORFs by homologous recombination. The diploid strain heterozygous for *ckb1*∆ and *pah1*∆ was obtained by mating after deletion of *CKB1* with a *NatMX* cassette in AWRI1631 and *PAH1* with a *KanMX* cassette in AWRI1633, which is a *MATα*, but otherwise isogenic derivative of AWRI1631. The diploid strain was sporulated as described above.

### Plasmid-based expressions

Acs1p bears a putative N-terminal signal for nuclear targeting and a peroxisome targeting-like signal (-VKL) at its C-terminal end. In addition, a lysine that was reported to be involved in the regulation of acetyl-CoA synthetase in *Salmonella enterica* through acetylation-dependent inactivation of the protein, is conserved in *S. cerevisiae* (K675). Full-length *ACS1* and a fragment encoding a N- and C-terminally truncated allele were amplified from genomic DNA. In addition, the reverse primer for the truncated variant was designed to introduce the K675R mutation. The plasmid pHEY-2 was obtained from pHEY-1 by replacing the *TEF1* promoter with the truncated *HXT7* promoter. This plasmid was linearized and assembled to pHEY-2/*ACS1* and pHEY-2/*ACS1*^*mut*^ by Gibson assembly with the respective PCR products.

For the overexpression of *ALD6*, *PLN1* and *Y. lipolytica OIL1*, the ORFs were amplified from genomic DNA and cloned into the vector pHEY-1 by Gibson assembly, resulting in the expression of the genes under the control of the strong constitutive *TEF1* promoter. Correct cloning was confirmed by sequencing.

ATP-citrate lyase of *Y. lipolytica* is a heterodimer, consisting of a regulatory and a catalytic subunit. Both subunits are required for activity. The genes encoding the two components of Acl were amplified from genomic DNA and cloned into the dual expression vector pSP-G1 [57]. Transformation of this vector resulted in expression of the regulatory subunit under control of the *TEF1* promoter and of the catalytic subunit under the control of the *PGK1* promoter.

In the yeast strains used for episomal expressions, *URA3* was deleted with a CRISPR/Cas9 strategy prior to transformation with the plasmids.

### Analytical methods

The CDW was determined after filtration of an aliquot of the culture through 0.45 µm cellulose nitrate filters and drying at 65 °C for 24 h.

For lipid analysis, culture volumes corresponding to ca. 15–30 mg CDW were centrifuged and the pellets were stored at -80 °C until extraction. The lipids were extracted, dissolved in 1 mL chloroform:methanol = 2:1 and analyzed by high performance thin layer chromatography according to [58]. As a control or for the determination of the FA composition, 100 µL of the extract were converted to FA methyl esters (FAMEs) according to [59] for the subsequent quantification by gas chromatography. The instrument was equipped with a flame ionization detector and analytical standards of the FAMEs of the four major FA in yeast (C16:0, C16:1, C18:0, C18:1) were used for calibration. C17:0 was used as an internal standard.

Furthermore, for some of the cultures of strains with high TAG content, culture aliquots corresponding to ca. 500 mg CDW were harvested as controls, to confirm the results obtained with the instrumental methods. The extracted lipids from these samples were weighed on an analytical balance to determine the lipid content of the strains.

For the measurement of the glycogen content, ca. 10^8^ cells were harvested. The glycogen was extracted and determined as described in [58].

For the determination of colony forming units after growth in nitrogen-limited medium, the culture density was determined with a Casy Cell Counter (Roche Innovatis) and diluted aliquots corresponding to 200 cells were spread onto YPD plates. The colonies were counted after two days of growth.

For the GAPDH assay, the strains were cultivated in 500 mL MM/C-lim until the late phase of exponential growth. The pellet was resuspended in 10 mL 10 mM Tris/HCl pH 7.4 + 0.5 mM EDTA and disrupted with glass beads. The cell debris was removed at 2500 g/3 min/4 °C and the cytosolic fraction was obtained as the supernatant after centrifugation for 45 min at 45,000 g/4 °C. This fraction was further purified by gel filtration and eluted with TEA buffer (350 mM triethanolamine, 30 mM Na_3_PO_4_, pH = 9.2), which was also used for the assay. The assay mix contained 200 µM NAD^+^ or NADP^+^, 1 mM glyceraldehyde-3-phosphate and an appropriate aliquot of the cytosolic fraction. 1 mM ADP was added to avoid equilibration of the reaction during the time of measurement (10 min at RT, 340 nM). The protein concentration of the fractions was determined with a BCA protein assay kit (Thermo Scientific) to calculate activities per mg total protein.

All data are the means from at least three independent experiments, performed on different days by plating aliquots from a frozen culture stock onto YPD plates, followed by cultivation and analysis as described above. The error bars represent standard errors. The p-values were calculated with an unpaired two-tailed t-test.

### Microscopy

For the staining of LD, culture aliquots were incubated for five minutes with Bodipy 493/503 (ThermoFisher). The images were taken on a Leica SP8 confocal microscope equipped with a 63× objective (NA 1.4), suited for immersion oil. Fluorescence of Bodipy was excited at 488 nm and emission detected in the range from 500 to 542 nm. The voltage of the photomultiplier was individually adjusted to the signal strength of the sample.

## Supplementary Information

**Additional file 1.** Additional tables and figures.

## Data Availability

All relevant data are within the manuscript and its Supporting Information file.
