# Supplementary material for: Engineering of Saccharomyces cerevisiae for the accumulation of high amounts of triacylglycerol
Source: Microb Cell Fact. 2021 Jul 27;20:147. doi: 10.1186/s12934-021-01640-0 (PMC8314538; doi:10.1186/s12934-021-01640-0)
Supplement: Supplementary file 1 — Additional file 1. Additional tables and figures. [file 12934_2021_1640_MOESM1_ESM.pdf]

Table S1: CRISPR/Cas9-based strategies, recognition sequences and repair DNA

	recognition sequence 5' -> 3'	repair DNA 5' -> 3'	resulting mutation
tgl3Δ	GCGTGTAGTGTATGCCACAT	CCCTTTTGAAAAACTGGATACTGCGTGTAGTGTATGCCACGACCATATACCTCCTTTTGTGTGGGAAATTCTACATGTCATC	frameshift
gsy1Δ	CCAGTCACTGTGGCACAATA	CTCTGTCTTGAAGTCCAAGGCTCCAGTCACTGTGGCACATACAGAGACAACTATACGCTGCTTGGACCTTTGAACAAGGCGAC	frameshift
gsy2Δ	TTTGTTATTCGAGACTGCGA	CCTCAGAGAAAAATTTTGAATGTCCCGTGACCTACAAAACAATTTTTATTCGAGACTGCGACTGAGGTTGCTAATAGGGTTGGTG	frameshift
are1Δ	CCTGCCACAGGAGTCCATGG	GTCACGTACGATAACGTGATCCTGCCACAGGAGTCCATGTAATTTCGCCACGGTCGTCTACCACGTCGCTGGTGGAGCC	frameshift
are2Δ	AACAATAACAAACACAACCA	CAGACACATTACGTTAGCAAAAGCAACAATAACAAACACAAGGGGGACAAGAAGAAGGATCTACTGGAGAACGAACAATTTCTCC	frameshift
sei1Δ	ATTGTAAAAACTGTAATGGA	ATAAGATAAAGTGAATAGGAAGGATGAAAATCAATGTATCTTACAGTTTTTACAATGGAGTTCATATATTGTTGTTGCATTTC	frameshift
ckb1Δ	TACAGGGTCCTCAGACGACG	CAAGAGTTTGTGGAAGACTATTCACGTACAGGGTCCTCAGACGAGATAGTGGAGCTTATGACGAGTGGATTCCATCGTTCTGTTCC	frameshift
ura3Δ	AGCTTGGCAGCAACAGGACT	AGCTACATATAAGGAACGTGCTGCTACTCATTAATAGTCCTGTTGCTGCCAAGCTATTTAATATCATGCACGAAAAGCAAACAAA	frameshift
ACC1	ATCTAAAATGGGTATGAACA	gttctccacctttccaactgttaaatctaaaatgggtatgaacaGAGCTGTTgctGTTTCAGATTTGTCATATGTTGCAAACAGTCAGTCATCTC<br><br>gttctccacctttccaactgttaaatctaaaatgggtatgaacaGAGCTGTTtctGTTTCAGATTTGTCATATGTTGCAAACAGTCAGTCATCTC	point mutation of Ser 1157 to Ala<br><br>point mutation of Ala 1157 to Ser
klGDP1^{co}	ACACACATAAACAAACAAAA	insertion of *klGDP1*^{co}	codon-optimized *klGDP1* under control of *TDH3* promoter; *tdh3Δ*
DGA1^{co}	TGAACAGTCACATAAATGTC	*TEF1*^{p}*-DGA1*^{co}	replacement of native *DGA1* with *DGA1*^{co} under control of the *TEF1* promoter
ChrI	GTATCACAACCGACGATCCG	*UTR1*^{co}	*UTR1*^{co} under control of the truncated *HXT7* promoter

Table S2: maximum specific growth rates during exponential growth and biomass and TAG yields after cultivation for 96 h in carbon-limited (MM/C-lim) or nitrogen-limited (MM/N-lim2) media for the wild-type and the engineered mutant 4/17-6 (*ACC1*$^{1157A\ 1257A}$ *tgl3*Δ *ckb1*Δ *are2*Δ *TEF1*^{P}-*DGA1*^{co} *gsy2*Δ).

	carbon-limited		nitrogen-limited	
	4/17	4/17-6	4/17	4/17-6
μ [h^{-1}]	0.36±0.02	0.34±0.01	0.36±0.03	0.33±0.02
CDW [g/L]	3.51±0.15	4.49±0.22	3.09±0.08	4.17±0.01
Y_{CDW}[1] [g/g]	0.18	0.22	0.15	0.21
TAG [mg/g_{CDW}]	161±6	593±7	223±6	654±14
Y_{TAG}[2] [g/g]	0.028	0.133	0.034	0.136

[1] biomass yield in g/g glucose

[2] TAG yield in g/g glucose

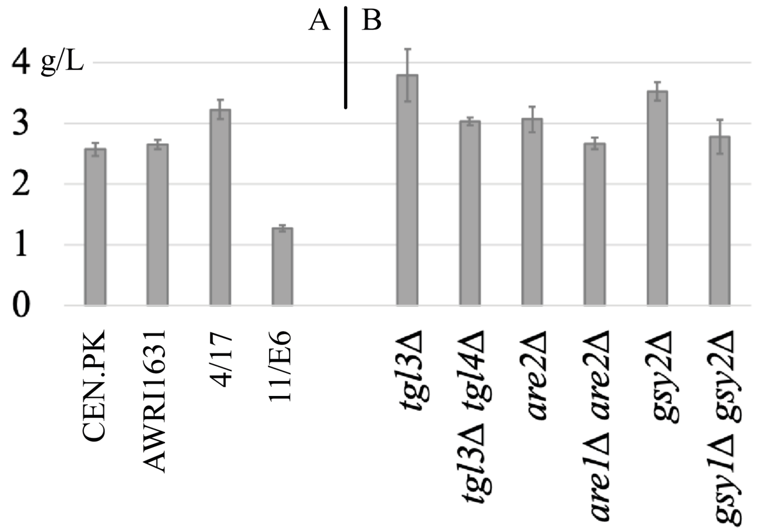

Figure S1: biomass yields after growth in MM/N-lim2, containing 20 g/L glucose and 0.6 g/L ammonium sulfate. A: Strain 4/17, one of the segregants from the sporulation of CEN.PKxAWRI1631, achieved a higher yield of biomass than the two parent strains, whereas the segregant with the highest TAG content, 11/E6 (derived from sporulation of BY4741xAWRI1631, see main text), had a growth defect and low final biomass. B: Double mutants in the competing pathways TAG lipolysis, SE synthesis and glycogen synthesis had a negative impact on the biomass yield.

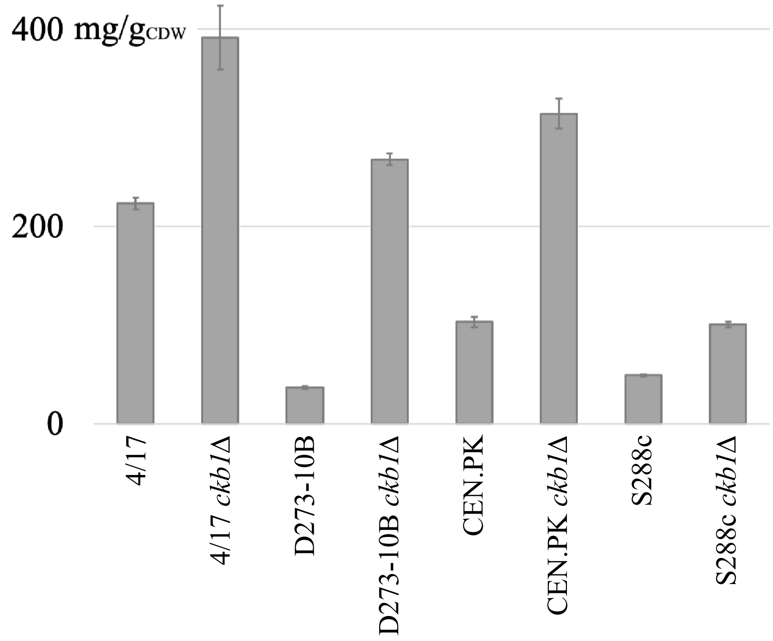

Figure S2: Deletion of *CKB1* in different strain backgrounds. *CKB1* was deleted in the three wild-type strains D273-10B, CEN.PK and S288c. The changes in TAG content suggest that the strong effect of this deletion is independent of the strain background.

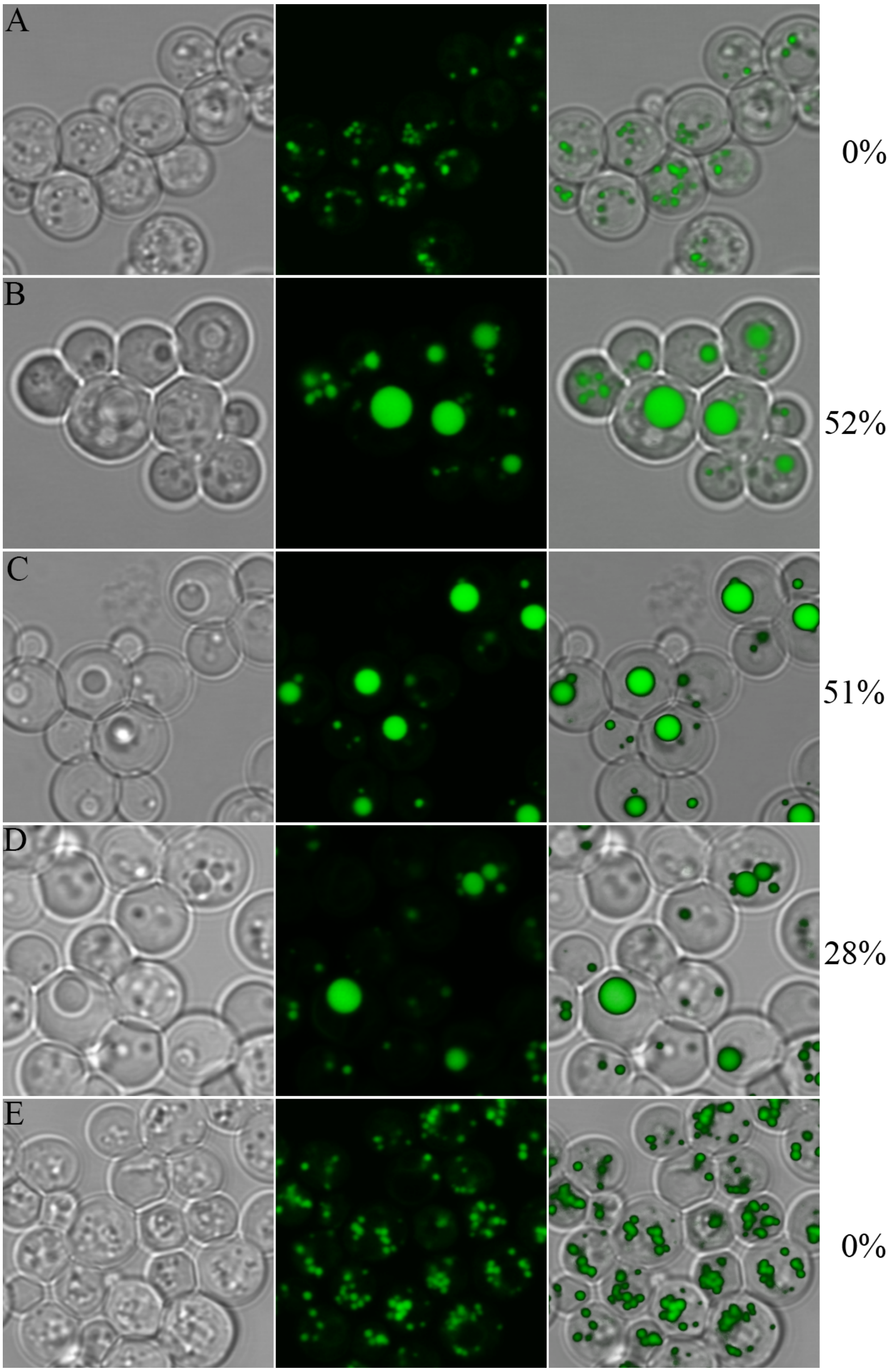

Figure S3: supersized LDs in *ckb1*Δ mutants. No supersizing of LDs was observed in the wild-type (A), whereas this phenotype was strong in the *ckb1*Δ mutant (B). The additional deletion of *GPT2* had no influence on supersizing (C). In contrast, the double mutant *nem1*Δ *ckb1*Δ resulted in a strain with less supersized LDs than the single mutant (D). The deletion of *SCT1* in the *ckb1*Δ background caused a complete loss of the supersizing phenotype.

The numbers indicate the percentage of cells with supersized LDs, determined by analyzing several hundred cells. The images show 20x20 µm representative sections of the strains during exponential growth in MM/C-lim. LDs were stained with Bodipy 493/503.

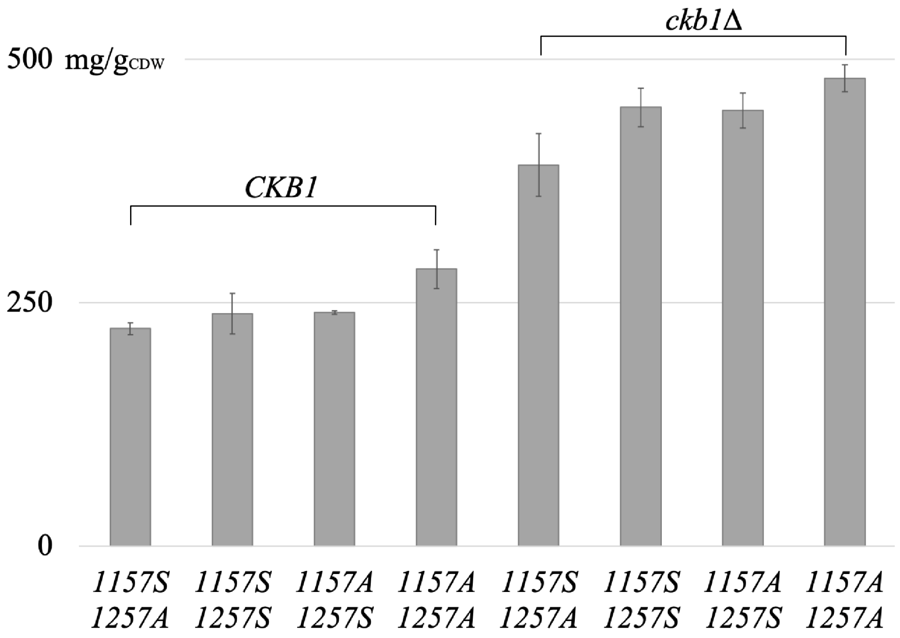

Figure S4: effect of the deletion of *CKB1* in combination with different *ACC1* alleles. *CKB1* was deleted in mutants of 4/17 with the four possible combinations of serine and alanine residues at positions 1157 and 1257 of Acc1p. The deletion of *CKB1* had a strong effect on TAG accumulation in all four backgrounds, independent of the presence of a serine residue at one of these positions.

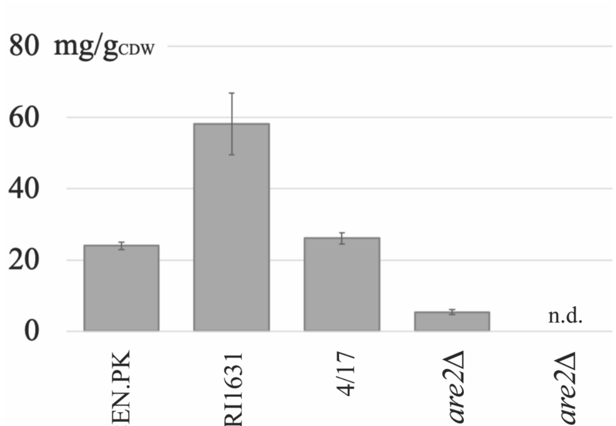

Figure S5: steryl ester storage. The figure shows the segregant with high lipid content, 4/17, and its two parent strains. The *are2*Δ and *are1*Δ *are2*Δ mutants were obtained by deletion of the respective genes in 4/17.

n.d. … not detected

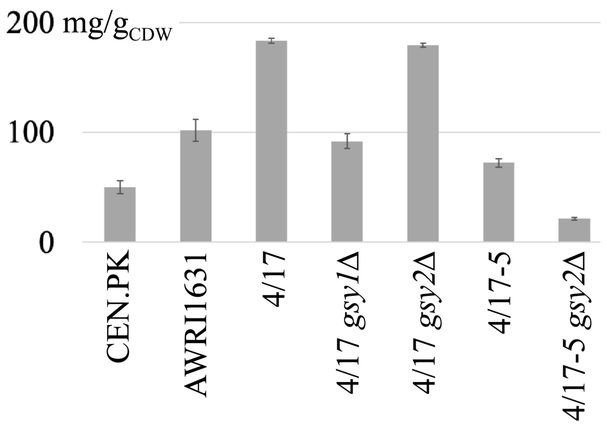

Figure S6: glycogen storage under nitrogen-limiting cultivation conditions. From left to right: parent strains CEN.PK and AWRI1631; segregant 4/17; deletions of *GSY1* and *GSY2* in the 4/17 wild-type background; segregant 4/17-5 (*ACC1*$^{1157A\ 1257A}$ *tgl3*Δ *ckb1*Δ *are2*Δ *TEF1*^{P}-*DGA1*^{co}) and the same strain bearing a deletion of *GSY2* (resulting in strain 4/17-6). Although the deletion of *GSY1* in the same background was viable in carbon-limited media, this strain could not be cultivated in nitrogen-limited medium.

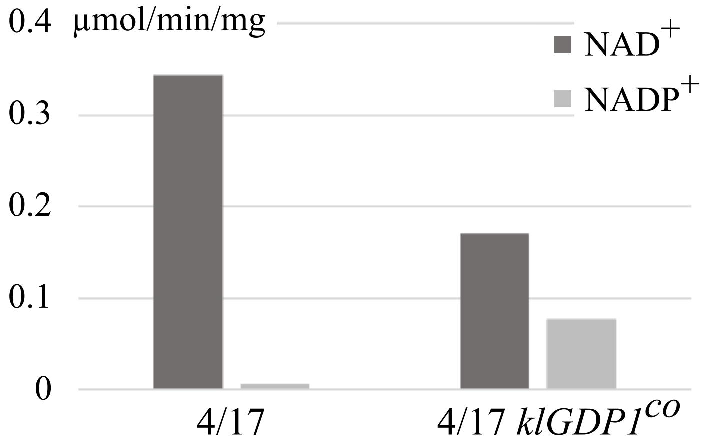

Figure S7: GAPDH activity in dependence of the cofactor. Segregant 4/17 was transformed with a codon-optimized ORF of *GDP1* from *K. lactis* that was inserted in the start region of the ORF coding for the major GAPDH of *S. cerevisiae*, *TDH3*. The total NAD$^+$-dependent *in vitro* activity in the mutant extract was less than 50% of the wild-type value, most likely due to the loss of *TDH3*. The NADP$^+$-dependent activity in the mutant, confirmed the expression of functional *klGDP1*^{co}.

The assays were performed with the cytosolic fractions after glass bead disruption. Therefore, the values represent activities per mg total cytosolic protein.

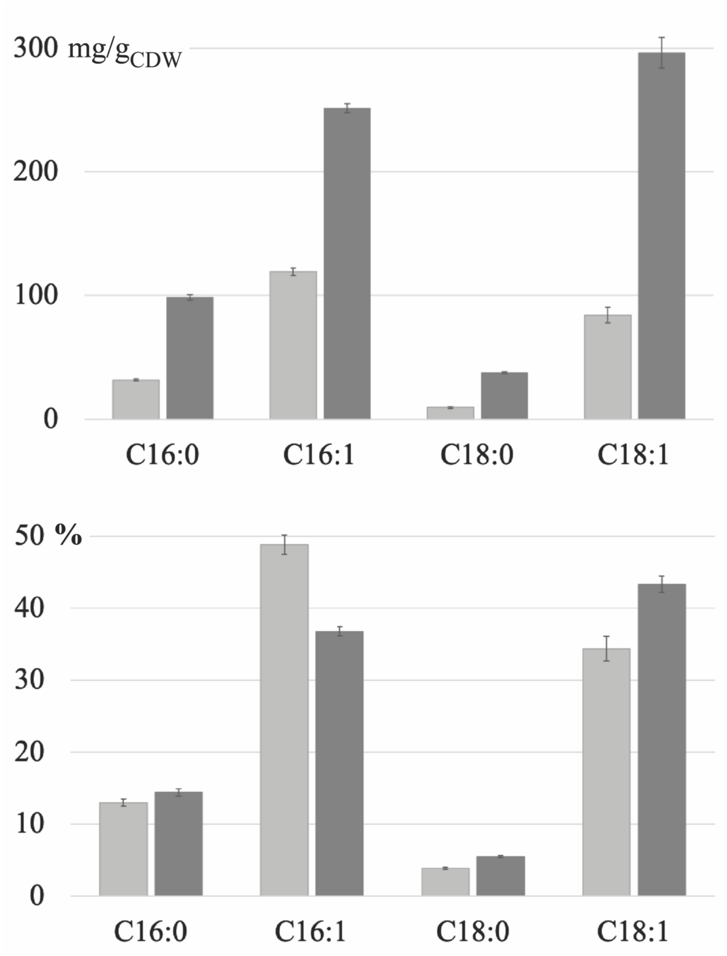

Figure S8: FAME analysis. The four major FA of the wild-type strain 4/17 (light gray) and of the strain engineered for high TAG content, 4/17-6 (dark gray), are shown as absolute values in mg FAME per g_{CDW} (upper panel) and as percentages (lower panel).

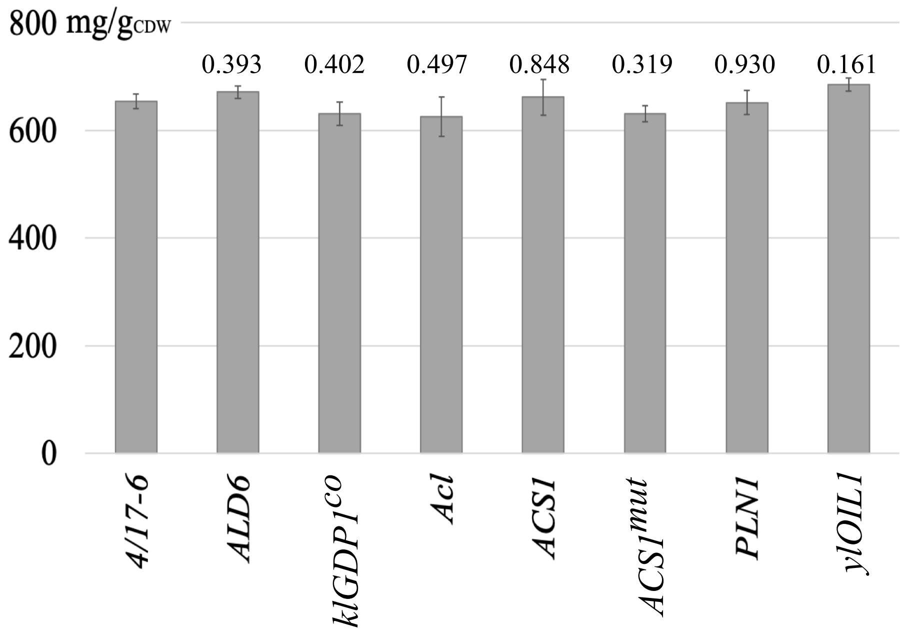

Figure S9: TAG content of the engineered strain 4/17-6, expressing the indicated genes. p-values above the columns are the results of a two-tailed t-test comparing the respective strain with the strain 4/17-6.
